# Association between fasting blood glucose and thyroid stimulating hormones and suicidal tendency and disease severity in patients with major depressive disorder

**DOI:** 10.17305/bjbms.2021.6754

**Published:** 2022-02-28

**Authors:** Weiting Liu, Zezhen Wu, Min Sun, Shuo Zhang, Juan Yuan, Dan Zhu, Guiming Yan, Kaijian Hou

**Affiliations:** 1 School of Nursing, Anhui University of Chinese Medicine, Hefei, China; 2 Department of Endocrine and Metabolic Diseases, Longhu Hospital, The First Affiliated Hospital of Medical College of Shantou University, Shantou, China; 3 College of Integrated Traditional Chinese and Western Medicine, Anhui University of Chinese Medicine, Hefei, China

**Keywords:** First-episode drug naïve, major depressive disorder, depression, thyroid hormones, blood glucose, diabetes mellitus, dyslipidemia, hypertension, suicidal behavior, suicidal attempts

## Abstract

Thyroid dysfunction and diabetes are reported to be associated with depression. However, their role in the suicide risk in patients with major depressive disorder (MDD) is unclear. The purpose of this study was to investigate and compare thyroid dysfunction and diabetes between suicide attempters and non-suicide attempters in a large sample of first-episode drug-naïve MDD patients. A descriptive study was conducted on 1279 Chinese outpatients with a diagnosis of first-episode MDD. Their socio-demographic information, blood levels of thyroid hormones, glucose, lipids and body mass index (BMI) parameters were collected. The positive subscales of the positive and negative syndrome scale (PANSS), Hamilton anxiety rating scale (HAMA), Hamilton depression rating scale (HAMD) were measured for psychotic, anxiety and depressive symptoms. Our results showed that compared with non-suicide attempters (*p < 0.01*), suicide attempters had statistically higher scores on HAMD, HAMA and PANSS psychotic symptoms, as well as higher thyroid stimulating hormone (TSH) serum levels, glucose, anti-thyroglobulin (A-TG), anti-thyroid peroxidase (A-TPO), total cholesterol (TC), triglycerides (TG), low density lipoprotein cholesterol (LDL-C), systolic blood pressure, and diastolic blood pressure (all with *p < 0.001*). These results revealed that TSH, A-TG, A-TPO, TC, TG, and LDL-C may be promising biomarkers of suicide risk in MDD, implying the importance of regular assessment of blood glucose level and thyroid function parameters for suicide prevention, along with possible treatment for impaired thyroid function and diabetes for the suicide intervention in MDD patients. Such patients with abnormal blood sugar and TSH must undergo thorough screening for suicidal ideation.

## INTRODUCTION

Depression, anxiety, and dementia are common psycho behavioral symptoms in autoimmune demyelinating multiple sclerosis (MS). The disturbance of reduction-oxidation homeostasis is commonly observed in MS. Monitoring various components of reactive chemical species, oxidative enzymes, antioxidative enzymes and degradation products, including kynurenines, was proposed to build personalized treatment plans for a better quality of life in MS [1].

The disturbance of lipid metabolism is gaining increasing attention in neuropsychiatric diseases and their comorbidities. A case-control study revealed that depression, diabetes mellitus and older age were associated with an increased likelihood of developing Alzheimer’s disease (AD), and dyslipidemia treatment reduced the likelihood of developing AD. The authors declared that depression and diabetes are risk factors of dementia, treatment of dyslipidemia reduces the risk of dementia, and aging is a decisive risk factor of dementia [2].

**FIGURE 1:**
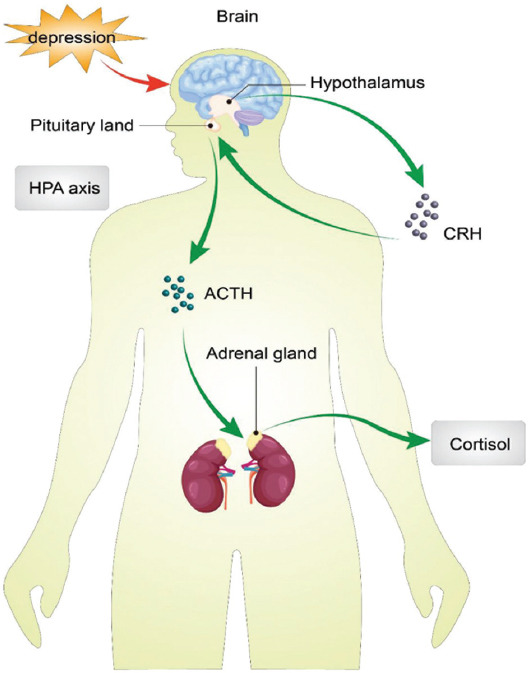
Hypothalamus-pituitary-adrenal axis (HPA axis) dysfunction is one of the important biochemical changes in depression. The development of depression will lead to changes in the function of HPA axis.

Major depressive disorder (MDD) is a highly prevalent psychiatric syndrome or psychopathological state observed in psychiatric clinics [3] that mostly occurs in adolescence and adulthood. The typical symptoms of MDD are mental retardation, depression and speech retardation or reduction. However, the pathogenesis and causes of MDD are complex. Recent studies have shown that its pathogenesis involves the imbalance of neuro-endocrine-immune network, in which the hypothalamus-pituitary-adrenal axis (HPA axis) plays a prominent role. It can interact with the nervous system and immune system to jointly affect the occurrence and development of depression [4]. If the patients are not treated reasonably in time, their mortality rate increases. Therefore, it is of great importance to study the factors related to the first-episode drug-naïve (FEDN) MDD (Figure 1).

In recent years, it has been known that diabetes, thyroid diseases and mental health diseases, including MDD, have shared etiology. Nearly, a quarter of patients with Type 2 diabetes have been diagnosed with depression, and the prevalence is much higher than that of non-diabetics [5]. At the same time, the incidence of thyroid disease is also higher in patients with MDD. Evidence suggests that abnormal thyroid function, especially hypothyroidism, is related to the severity and obvious psychopathological characteristics of depression [6]. The risk of incident diabetes mellitus was higher among subjects with depression compared with non-depressed subjects. The estimated rate of diabetes mellitus attributable to depression was 6.87% [7]. Diabetes can significantly increase the suicide probability of MDD patients [8]. Studies have shown that the dysfunction of serotoninergic (5-hydroxytryptamin [5-HT]) and hypothalamus-pituitary-adrenal cortex axis can predict the risk of suicide [9]. It affects the serum thyroid-stimulating hormone (TSH) level by regulating the growth and maturation of certain brain regions during the brain development. The risk of suicide is significantly higher in patients with depression than that of most other diseases [8], and the risk of suicide is particularly higher in patients with MDD. Among MDD patients, if there are abnormal blood sugar and/or TSH levels, the risk of suicide attempts increases. Based on these observations, MDD patients with abnormal blood glucose and TSH levels should be required to undergo a thorough suicidal ideation screening. At present, most suicidal behaviors can only be assessed by scales, and many high-risk groups are unwilling to disclose their suicidal ideas or plans due to the fear of discrimination, forced hospitalization, or angst that their suicide plan will be hindered by others. The evaluation results are unreliable. Therefore, the combination of internal and subjective scale evaluation and external and objective biological testing can more accurately evaluate suicidal behavior. It is particularly important to establish an objective examination method for the diagnosis of MDD, especially suicide attempts.

To identify some potential risk factors for the severity of MDD patients and reduce the incidence of MDD, we tested the levels of thyroid hormones, blood glucose, blood lipids and body mass index (BMI) in 1279 Chinese patients with MDD in the first stage, then analyzed the correlation between these factors and the patient’s condition.

Despite growing interest in the psychiatric implications of thyroid dysfunction and diabetes, most published studies still focus on their association. A further investigation of this association has importance for the public heathland patients. Societal and economic costs caused by depression and anxiety disorders are high [10,11] and can be lowered by an appropriate and early treatment. By quantitatively summarizing, the results concerning thyroid hormones and blood glucose level as biomarkers of MDDs, the awareness of this association will increase and appropriate thyroid, antidiabetic and antidepressant treatment can be implemented beyond psychotherapy. Furthermore, screening tests for symptoms of depression and anxiety in patients associated with thyroid dysfunction and diabetes could be implemented. The aim of the present study was to estimate the association and prevalence of thyroid dysfunction and diabetes in patients with MDD. Furthermore, we aimed at identifying potential clinical and socio-demographic characteristics of MDD patients associated with thyroid dysfunction and diabetes.

## MATERIALS AND METHODS

### Study design and setting

This study was conducted at Endocrine Department, Longhu Hospital China during the period of August 2019-January 2020.

### Study subjects

The study enrolled 1279 patients from the Endocrine Department, Longhu Hospital China during the period of August 2019-January 2020 that were diagnosed with major depression disorder. Only patients who met the following criteria were included: (1) Patients meeting the diagnosis of MDD based on the Diagnostic and Statistical Manual of Mental Disorders, Fourth Edition; (2) Patients used no drugs related to thyroid metabolism within 30 days before enrollment; (3) Patients were 17-60 years old and had scored 24-41 on the Hamilton Depression Scale. Patients who met the following criteria were excluded: (1) Patients with hypomanic episode and maniacalis insultus; (2) Substance-dependent patients and other patients with mental illness; (3) Patients with severe physical illness and endocrine diseases; (4) Patients in lactation and pregnancy; (5) patients with post-traumatic stress disorder.

The patients with first-episode MDD included 440 males and 839 females, with an average age of 35.13 ± 10.13 years. After hospital admission, balanced diets were provided daily for the patients, and all patients had 1 hour of physical exercise every day. This study was approved by the ethical approval number JXSQ202101. All the patients and their families were informed and signed informed consents.

### Sample size

The sample size of the study was calculated taking reference to the previously published study, which reported 22% prevalence of thyroid disorder among depressive patients [12] using the formula:

Sample size (X) = Zα/22 *p*(1-p)/MOE2

where:

n = sample size

Z = 1.96 at 95% CI.

p = prevalence of thyroid disorder among depressive [12]

e = margin of error (5%)

### Hypothesis

We hypothesized that patients with thyroid dysfunction and diabetes have a substantially higher risk of developing suicidal attempts and disease severity in patients with MDD.

### Clinical measurement

A general situation questionnaire was used to collect the clinical and demographic data of the subjects. The Hamilton Depression Scale (HAMD), Hamilton anxiety scale (HAMA), and positive and negative syndrome scale (PANSS) were used to evaluate the psychotic (neuro) symptoms (neuro) psychological symptoms associated with the attentional and executive disturbances [13], fatigue [14], or reduction in quality of life [15,16] of patients. Two senior clinicians evaluated the positive and negative symptoms of the patients in the study.

### Measurement of thyroid hormone, blood glucose, blood lipid, and BMI

After admission, all patients were fasting before the morning when the peripheral blood was collected. The peripheral blood was placed in a coagulation tube for 30 minutes, and then centrifuged at 3000 rpm for 15 minutes. The serum was extracted and stored at –40°C.

The levels of TSH, anti-thyroglobulin (A-TG), anti-thyroid peroxidase (A-TPO), Free triiodothyronine, and Free thyroxine in serum samples were measured by an electrochemiluminescence immunoassay kit (Roche, Germany) according to the manufacturer’s directions (Roche, Germany). At the same time, the age, sex, weight, height, blood pressure (diastolic and systolic blood pressure), BMI, blood lipid levels (total cholesterol [TC], triglycerides [TG], HDL-C, low density lipoprotein cholesterol [LDL-C]), fasting blood glucose levels, and 1 hour postprandial blood glucose levels were collected.

### Statistical analysis

The data analysis was performed using the IBM SPSS 20.0 statistical software. The measurement data were represented by mean ± standard deviation. The t-test was used to compare the intra- and inter-group of the observation group and the control group, while the count data were detected by chi-square test. *p < 0.05* was considered statistically significant.

## RESULTS

### The total positive score

The total positive scores (PANSS scores) of MDD patients were correlated with thyroid hormone, blood glucose, blood lipid and blood pressure, but not with age, gender, course of the disease, age of disease onset, educational background, marital status, nor BMI and FT4 (Table 1). The levels of TSH, A-TG, A-TPO, fasting blood glucose, TC, TG, LDL-C and blood pressure in MDD patients with total positive scores ≥8 points were significantly higher than those in MDD patients, with total positive scores of 7 points, while HDL-C in MDD patients with total positive scores ≥8 points was significantly lower than that in MDD patients with total positive scores of 7 points. The differences between the above results were all statistically significant (*p < 0.05*).

**TABLE 1:**
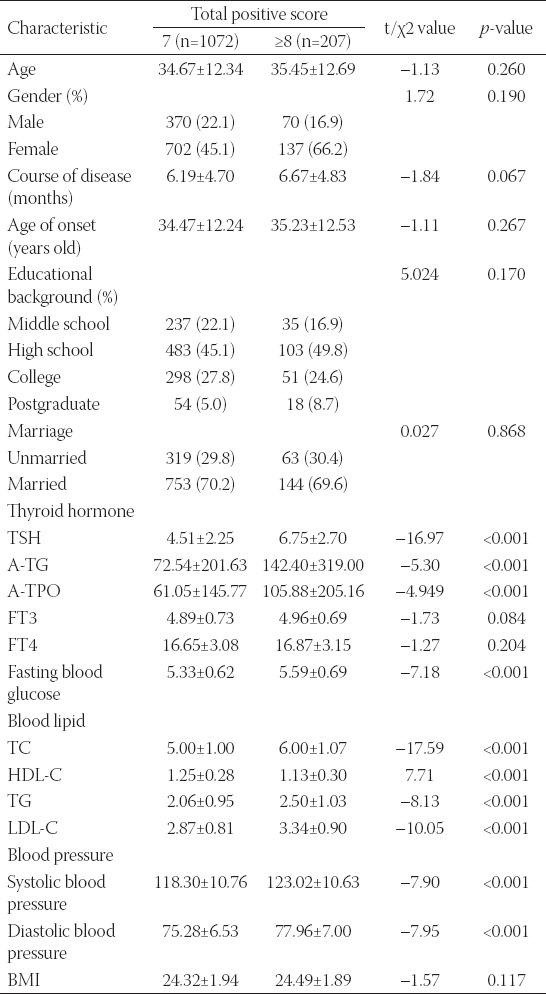
The relationship between total positive score and clinical characteristics

### Association between suicidal behavior and the condition of MDD patients

As shown in Table 2, the suicidal behavior in MDD patients was correlated with age, age of disease onset, course of the disease, education, thyroid hormones, blood glucose, blood lipids and blood pressure, but not with gender, marital status, nor BMI (*p > 0.05*). The age of patients with suicidal behavior was significantly higher than that of patients without suicidal behavior, with a significant difference (*p < 0.05*). Moreover, the MDD patients with suicidal behavior had relatively late-onset, long duration and low academic qualification, which showed a statistically significant difference (*p < 0.05*). TSH, A-TG, A-TPO, fasting blood glucose, TC, TG, LDL-C, systolic blood pressure and diastolic blood pressure were all obviously higher in MDD patients with suicidal behavior when compared with those of patients without any suicidal behavior, and the difference was statically significant (*p < 0.01*). In contrast, the HDL-C level of MDD patients with suicidal behavior was evidently lower than that of patients without suicidal behavior (*p < 0.001*). The difference was also statistically significant.

**TABLE 2:**
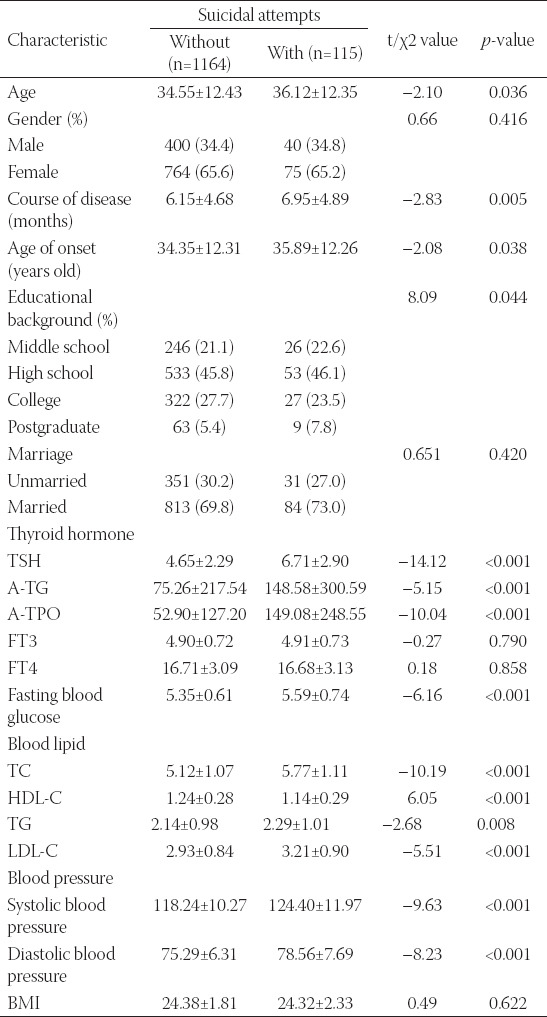
Analysis of variable-difference tests in patients with and without suicidal attempts

To the best of our knowledge, this study compared the thyroid function of suicide and non-suicide attempters in FEDN MDD for the 1^st^ time in a large sample size. Our study shows that suicide attempts are associated with increased serum levels of TSH, TGAB and TPOAB, which suggests that these biomarkers could be used for evaluating suicide attempts in patients with MDD. In addition, we found that after controlling for demographics, anxiety, depression and psychiatric symptoms, only TPOAB remained an independent risk factor for suicide attempts and the OR value was reduced. This means that high levels of TSH and TGAB may increase the risk of suicide by exacerbating symptoms of anxiety, depression and psychosis, while TPOAB may increase the risk of suicide and exacerbate these symptoms (Table 2).

### Association between severe anxiety and the condition of MDD patients

As shown in Table 3, the presence or absence of severe anxiety in MDD patients was correlated with age, duration of disease, age of onset, marital status, thyroid hormone, blood glucose, blood lipids, blood pressure and BMI, but not with gender, and there was no statistically significant difference found for any of these indices. In addition, the age of MDD patients with severe anxiety symptoms was significantly higher than that of patients without severe anxiety symptoms, and the difference was statistically significant (*p < 0.05*). MDD patients with severe anxiety symptoms were characterized by the late-onset, longer duration and lower education (*p < 0.05*). Married depressed patients had a higher tendency for severe anxiety than unmarried MDD patients (*p < 0.05*). Furthermore, the TSH, A-TG, A-TPO, fasting blood glucose, TC, TG, LDL-C, systolic blood pressure, diastolic blood pressure, and BMI were all dramatically higher in patients with severe anxiety symptoms than in patients without severe anxiety (*p < 0.05*). HDL-C was significantly lower in patients with severe anxiety than those without severe anxiety (*p < 0.001*).

**TABLE 3:**
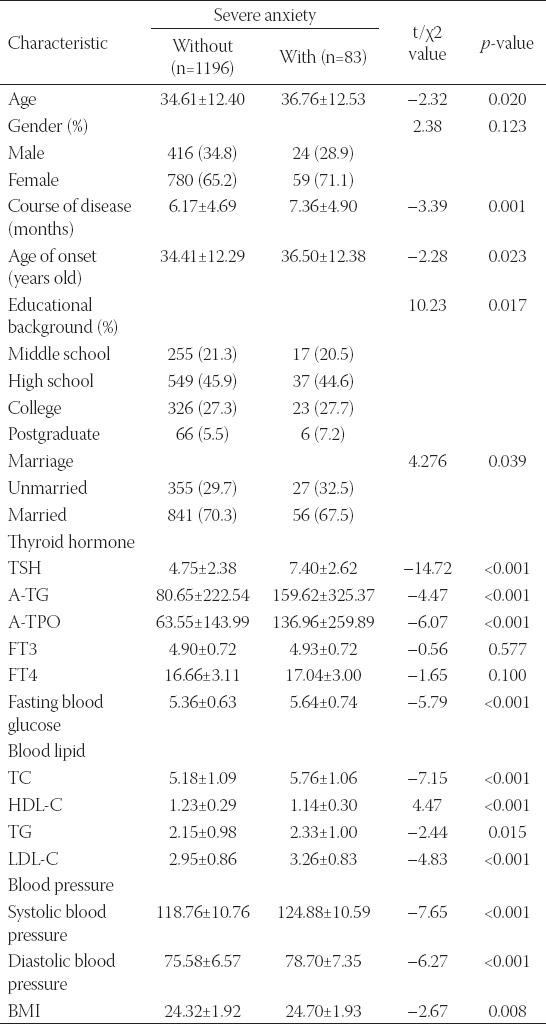
Analysis of variable-difference tests in patients with and without severe anxiety

### Association of psychotic symptoms in MDD patients

The presence of psychotic symptoms in MDD patients was related to age, age of onset, thyroid hormone, blood glucose, blood lipid, blood pressure and BMI, but not to gender, course of the disease and marital status. MDD patients with psychiatric symptoms were older than those without psychiatric symptoms (*p<0.05*). MDD patients with psychiatric symptoms had the characteristics of later onset age and lower education level than those without psychiatric symptoms (*p<0.05*). There was no significant difference in severe anxiety between Married and Unmarried MDD patients. The TSH, A-TG, A-TPO, fasting blood glucose, TC, TG, LDL-C (*p<0.05*), systolic blood pressure, diastolic blood pressure, and BMI of depressive patients with psychiatric symptoms were significantly higher than in those without psychiatric symptoms (*p<0.05*). However, the HDL-C of depressive patients with psychiatric symptoms was significantly lower than in those without psychiatric symptoms (*p=0.001*), as shown in Table 4.

**TABLE 4:**
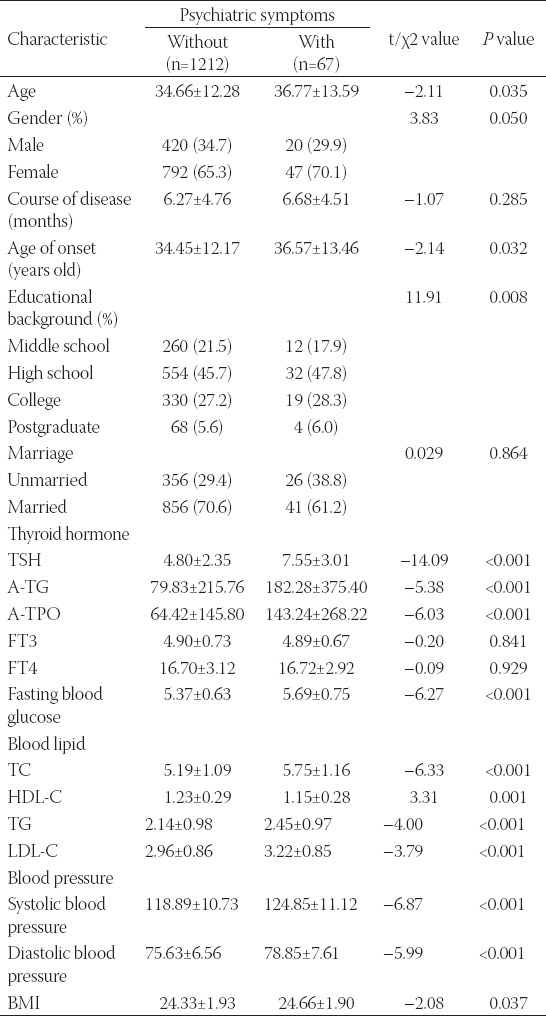
Analysis of variable-difference tests in patients with and without psychiatric symptoms

## DISCUSSION

MDD is a common mental illness that has been predicted to be the main cause of disability in the world [17]. In general, the HAMD and HAMA scores can reflect the severity of MDD and predict the occurrence of suicide attempts in patients [18]. The positive subscale of PANSS can be used to evaluate the mental symptoms of patients with major depression [19], and compare them with depressed patients without severe mental disorders. Patients with severe mental disorders usually have suicide attempts [20]. The results here show that the depressive symptoms, severe anxiety, PANSS total score and suicidal behavior of the first-episode MDD patients in China are related to thyroid hormones, blood sugar, blood lipids levels, as well as blood pressure, indicating that metabolic disorders of sugar, lipids and thyroid hormones are closely related to the development of major depression.

The serum levels of thyroid hormones are closely related to the occurrence and development of mental illness [21]. Experiments showed that the dysfunction of the dorsal nucleus and nucleus lifts by the transverse pine habenula dysfunction reduces the regulation of serotonin levels. Reduced thyroid hormone levels promote depression symptoms in MDD rats [21]. Patients with depression may have various thyroid abnormalities, including excessive or insufficient thyroid hormone levels [22]. Consistently, the results of Ittlermann et al. [23] and others indicate that the abnormal levels of thyroid hormones in patients with MDD and anxiety are associated, which is related to the existence of depression and anxiety, while thyroid peroxidase antibodies are associated with characteristic signs of depression [24].

When patients with depression are in a state of depression for a long time, the body’s 5-HT, acetylcholine, norepinephrine and other hormone concentrations decrease. At this time, thyroid hormones play a compensatory role to prevent the further development of depression. However, in a long-term depression, especially for patients with major depression, the thyroid gland might lose its compensation and hypothyroidism will result. TSH is significantly associated with psychotic symptoms, anxiety and depression. TSH may increase the risk of suicide by aggravating anxiety, depression, and psychotic symptoms [24].

This study found that MDD patients with hypothyroidism are more anxious and depressed than MDD patients with euthyroidism, and even have suicidal behaviors. TSH values are significantly higher, indicating that TSH may be a promising biomarker for evaluating suicide attempts in MDD patients.

In fact, lipid metabolism disorders can also be risk factors for the development of MDD. Our results show that the total positive score of TG and LDL-C levels ≥8 points in MDD patients is significantly higher than that of MDD patients, and the total positive score is <7 points. In agreement with our research results, some previous studies have shown that low-density lipoprotein-C levels can significantly affect the incidence of MDD in postmenopausal women [25]. In addition, Hidese et al. [26] revealed a positive correlation between the condition of MDD patients and blood lipid levels in a Japanese population study.

Interestingly, we found that the suicidal behavior of Chinese patients with MDD in the first phase of this study is related to blood lipid levels. A possible mechanism can be postulated. The decrease in serum cholesterol levels may lead to a decrease in brain cell membrane cholesterol, thereby reducing the microviscosity of brain cell membrane lipids. This may result in the exposure of the serotonin receptor protein to the membrane surface, due to the reduction of serotonin reabsorption in the blood. Subsequently, the surface location of the serotonin receptor affects the entry of serotonin into human brain cells. Here, the combination of the effect of serotonin on impulse control in the brain and the reduction of blood cholesterol levels may lead to suicide and violent behavior in susceptible people [27].

In this study, the total positive score (PANSS score), suicidal behavior, severe anxiety and psychiatric symptoms of Chinese patients with MDD in the first stage were positively associated with blood glucose levels, and glucose metabolism disorders are believed to accelerate the development of MDD. According to the reports, the relative risk of diabetes in patients with depression is 1.2-2.6 times that of patients with non-depression [28]. MDD patients may have varying degrees of abnormal glucose metabolism, including elevated fasting blood glucose, insulin and glucagon levels [29]. This study further found that compared with MDD patients without suicidal behavior, MDD patients with suicidal behavior had higher fasting blood glucose levels. In MDD patients, a significant correlation was found between suicidal behavior and fasting blood glucose levels (*p* < 0.001), where symptoms of depression and anxiety may increase the risk of suicide by raising blood sugar levels. Depression is related to poor blood sugar control [30] and high HbA1c [31]. The incidence of MDD in Type 2 diabetes patients is significantly higher than that in the gender and age-matched population, and it is closely correlated with the increase of age at diagnosis [32]. Anxiety and depression will increase the risk of diabetes [33]. The correlation between blood sugar levels and depression symptoms is related to metabolic factors, signaling pathways, genetic factors and many other factors. The loss of serotonin activity in the brain of diabetic patients is related to depression behavior. Serotonin receptor antagonists have the effect of promoting serotonin response. Therefore, understanding the role of the serotonin pathway in MDD may provide treatment options in the future, and the development of new clinical antidepressants will pave the way [34,35].

Our analysis of suicidal behavior showed that the TSH, fasting blood glucose and TC serum levels of suicidal MDD patients were higher than those of MDD patients without suicidal behavior, which was statistically significant. We believe that fasting blood glucose and TSH can also be used to assess the severity of illness in MDD patients and as biomarkers for suicide attempts. The study by Koponen et al. [36] also observed that patients who had suicidal ideation or had attempted suicide had higher blood glucose levels in OGTT (Oral glucose tolerance test), which is a glucose load test to understand the function of pancreatic β-cells and the body’s ability to regulate blood sugar. It is a diagnostic test for diagnosing diabetes at baseline and 2 hour sampling.

Studies have shown that compared with MDD patients without anxiety symptoms, MDD patients with anxiety symptoms have a significantly higher incidence of suicide attempts and psychiatric symptoms [18], which is also more consistent with our research results. Other studies have shown [37] that mental disorders including MDD may change the composition of the intestinal flora. In the past 10 years, the field of the intestinal flora of MDD patients has attracted much attention. In observational studies, compared with non-depressed controls, MDD patients had significantly reduced intestinal flora at the level of family and genus. In the intervention study using probiotics, compared with the control group, the symptoms of depression were significantly improved. Some neurotransmitters produced by the gut microbiota, such as GABA and serotonin, may also be related to the risk of neuropsychiatric diseases, indicating that they play an important role in microbial-host interactions in brain function and behavior. On the other hand, mental disorders including AD and MDD may change the composition of the intestinal flora [37]. The changes in the intestinal flora and the impact of these changes on MDD patients and suicidal behaviors remain to be explored further.

The limitations of our research include the following: First, this research is a cross-sectional study, which focuses on the investigation of the cause of the disease, and it is temporarily unable to draw a causal relationship; second, this study did not set up a control group of healthy people, nor did it consider the influence of factors such as environment, income, stress and others on suicidal behavior; Third, this study does not consider undiscovered comorbid factors that promote suicidal behavior in MDD patients, thus ignoring other factors that trigger suicidal behavior in MDD patients.

## CONCLUSION

In summary, this study shows that factors such as thyroid hormones, blood sugar, blood lipids, and BMI index can aggravate MDD and suicidal behavior. We found that MDD patients with suicidal behaviors had higher TSH and fasting blood glucose levels than MDD patients without suicidal behaviors, and the difference was statistically significant. Therefore, we believe that patients with MDD may need to monitor their thyroid function and blood sugar levels regularly after diagnosis, to detect potential thyroid and blood sugar abnormalities in time, to prevent the risk of suicidal behavior. In addition, TSH and fasting blood glucose levels could be used as screening indices for major depression, to assess the tendency of suicidal behavior and to help patients with depression get timely treatment.
